# Functional Overload Enhances Satellite Cell Properties in Skeletal Muscle

**DOI:** 10.1155/2016/7619418

**Published:** 2015-12-08

**Authors:** Shin Fujimaki, Masanao Machida, Tamami Wakabayashi, Makoto Asashima, Tohru Takemasa, Tomoko Kuwabara

**Affiliations:** ^1^Stem Cell Engineering Research Group, Biotechnology Research Institute for Drug Discovery, Department of Life Science and Biotechnology, National Institute of Advanced Industrial Science and Technology (AIST), Central 4, 1-1-4 Higashi, Tsukuba Science City, Ibaraki 305-8562, Japan; ^2^Physical Education, Health and Sport Sciences, Graduate School of Comprehensive Human Sciences, University of Tsukuba, 1-1-1 Tennodai, Tsukuba Science City, Ibaraki 305-8574, Japan; ^3^Organization for General Education, Saga University, 1 Honjo-machi, Saga 840-8502, Japan

## Abstract

Skeletal muscle represents a plentiful and accessible source of adult stem cells. Skeletal-muscle-derived stem cells, termed satellite cells, play essential roles in postnatal growth, maintenance, repair, and regeneration of skeletal muscle. Although it is well known that the number of satellite cells increases following physical exercise, functional alterations in satellite cells such as proliferative capacity and differentiation efficiency following exercise and their molecular mechanisms remain unclear. Here, we found that functional overload, which is widely used to model resistance exercise, causes skeletal muscle hypertrophy and converts satellite cells from quiescent state to activated state. Our analysis showed that functional overload induces the expression of MyoD in satellite cells and enhances the proliferative capacity and differentiation potential of these cells. The changes in satellite cell properties coincided with the inactivation of Notch signaling and the activation of Wnt signaling and likely involve modulation by transcription factors of the Sox family. These results indicate the effects of resistance exercise on the regulation of satellite cells and provide insight into the molecular mechanism of satellite cell activation following physical exercise.

## 1. Introduction

Skeletal-muscle-specific stem cells, termed satellite cells, contribute to the postnatal maintenance, growth, repair, and regeneration of skeletal muscle [1]. These cells are located between the basal lamina and plasma membrane of skeletal muscle fibers in which they represent 2.5%–6% of all nuclei and remain in a quiescent state under normal physiological conditions [2]. In response to muscle injury or exercise, satellite cells are activated and proliferate and differentiate into mature fibers [3]. Exercise positively affects muscle fiber composition via regulation of satellite cells to improve muscle performance. Previous studies have shown that the number of satellite cells is increased by long-term or acute exercise training in humans and animals [4, 5] and decreases during aging in conjunction with a reduction in the muscle quality and functional potential [6]. Loss of skeletal muscle mass, known as sarcopenia, is a serious health issue that affects millions of aging adults. Since exercise can improve muscle strength and endurance capacity, it can serve as a means of preventing muscle atrophy and reducing the risk of sarcopenia.

Satellite cells can be mitotically quiescent or in an activated proliferative state during skeletal muscle turnover. These two states can be distinguished by the expression of specific markers. All satellite cells express the stem-cell-specific transcription factor, paired-box 7 (Pax7). In addition, activated satellite cells express myogenic factor 5 (Myf5) and myogenic differentiation (MyoD) [7]. There have been few studies examining functional alterations in satellite cells such as proliferative capacity and differentiation efficiency following exercise. Furthermore, the molecular mechanisms by which exercise-stimulating extracellular factors control the satellite cell activation and differentiation remain unclear.

Physical exercise induces changes in extracellular signaling in skeletal muscle that affect satellite cells. For instance, Notch signaling is involved in fate determination and regulates satellite cell proliferation, and previous studies have shown that physical exercise increases the expression of Notch signaling pathway components—including ligands, Notch receptor, and downstream effectors—in myogenic cells [8–10]. On the other hand, Wnt signaling, which contributes to satellite cell activation and lineage specification in skeletal muscle, is activated by exercise [11–13]. The shift from Notch to Wnt signaling controls the transition from proliferation to differentiation in myogenic progenitors during muscle regeneration [14]. Although the effect of exercise on Notch and Wnt signaling has been well studied, detailed knowledge of their relationship to satellite cell function remains elusive.

Functional overload (FO) is experimentally induced by ablating of synergistic muscles in the facies posterior of the lower legs of animals and is widely used to model resistance exercise, leading to a variety of physiological effects such as skeletal muscle hypertrophy and metabolic improvement as well as muscle fiber-type transition [15–18]. Notably, the number of satellite cells in skeletal muscle increases following FO by mechanisms that are as yet unclear [19].

In this study, we investigated the effects of FO on satellite cells, including their proliferation and differentiation. We found that muscle mass and the number of activated but not of quiescent satellite cells increased following FO, which also increased the proliferative capacity and differentiation potential of these cells. Changes in satellite cell properties were accompanied by the inactivation of Notch signaling and the activation of Wnt signaling. These results provide insight into the molecular mechanism of satellite cell activation following physical exercise.

## 2. Methods

### 2.1. Animals

Animal experiments were carried out in a humane manner after receiving approval from the Institutional Animal Care and Use Committee of the National Institute of Advanced Industrial Science and Technology. Animals were housed in standard cages in facilities with controlled temperature and humidity under a 12 : 12 h light/dark cycle and had free access to chow and water. Female Fischer344 rats (Japan SLC Inc., Hamamatsu, Japan) 12 weeks of age were used in this study. Rats were randomly divided into control and FO groups. There were no differences in body weight among rats at the start of the experiment.

### 2.2. FO and Tissue Sampling

The plantaris muscle of rats in the FO group was overloaded by surgically removing the soleus and gastrocnemius muscles as previously described [15]. Rats were sacrificed 2 weeks after the surgery with an overdose of pentobarbital. For RNA or protein extraction, the plantaris muscle was dissected from each rat and frozen in liquid nitrogen after measuring the wet weight and stored at −80°C until homogenization. For immunohistochemistry, rats were subjected to transcardial perfusion with phosphate-buffered saline followed by 4% paraformaldehyde (PFA). The plantaris muscle was dissected and postfixed in 4% PFA until analysis.

### 2.3. Satellite Cell Isolation and Culture

Primary satellite cells were obtained from plantaris muscles digested with pronase as previously described [20]. To assess proliferation, cells were cultured in growth medium consisting of low-glucose Dulbecco's Modified Eagle's Medium (DMEM) supplemented with 10% fetal bovine serum (FBS), 20 ng/mL basic fibroblast growth factor (FGF), and 1% antibiotic-antimycotic liquid (anti-anti) in laminin-coated 8-well chambers at 37°C and 5% CO_2_. The medium was removed 3 days later and cells were fixed with 4% PFA for immunocytochemistry. To evaluate satellite cell differentiation, myogenic differentiation was induced by culturing cells in differentiation medium consisting of low-glucose DMEM supplemented with 10% FBS, 10% horse serum, and 1% anti-anti in laminin-coated 6-well plates or 8-well chambers at 37°C and 5% CO_2_. After 1 and 2 days, cells in the 8-well chambers were fixed with 4% PFA for immunocytochemistry and those in the 6-well plates were collected in Isogen reagent (Nippon Gene, Tokyo, Japan) for RNA isolation.

### 2.4. Immunostaining

Fixed samples were placed in a 30% sucrose solution and stored at 4°C overnight. Cross sections were cut at a thickness of 200 *µ*m on a microtome (ROM-380, Yamato Kohki, Saitama, Japan) and stored in tissue collection medium (25% glycerin, 30% ethylene glycol, and 0.05 M PO_4_) at −20°C until analysis. Sections and fixed cells were washed, permeabilized with Tris-buffered saline (TBS) containing 0.25% Triton X, blocked with 5% normal donkey serum in TBS, and incubated with the following primary antibodies: mouse anti-Pax7 (1 : 10; Developmental Studies Hybridoma Bank (DSHB), Iowa City, IA, USA), rabbit anti-MyoD (1 : 200; Santa Cruz Biotechnology, Santa Cruz, CA, USA), rabbit anti-Dystrophin (1 : 200; GeneTex, Irvine, CA, USA), and mouse anti-myosin heavy chain (MyHC) (MF20, 1 : 10; DSHB) for 3 days (sections) or 1 day (cells) at 4°C. Immunoreactivity was detected by incubation with Cy3-conjugated donkey anti-mouse IgG (1 : 500; Jackson ImmunoResearch, West Grove, PA, USA) or Alexa Fluor 488-conjugated donkey anti-rabbit IgG (1 : 500; Life Technologies, Carlsbad, CA, USA) overnight at 4°C. Samples were counterstained with 4′,6-diamidino-2-phenylindole (Wako Pure Chemical Industries, Osaka, Japan). After several washes, sections and cells were mounted on glass slides using Vectashield (Vector Laboratories, Burlingame, CA, USA). Images were acquired using an Olympus FV1000-D confocal microscope (Olympus, Tokyo, Japan).

### 2.5. RNA Isolation and Quantitative Real Time- (qRT-) PCR Analysis

Total RNA was isolated from frozen muscle tissue and cultured cells using Isogen reagent. RNA samples were treated with Turbo DNase (Life Technologies, Carlsbad, CA, USA) to remove genomic DNA. cDNA was synthesized using PrimeScript RT Master Mix (Takara Bio, Otsu, Japan) according to the manufacturer's recommendations. The cDNA was diluted 10-fold with diethylpyrocarbonate-treated water and used as a template for qRT-PCR, which was carried out using Thunderbird SYBR qPCR Mix (Toyobo, Osaka, Japan) on a CFX96 system (Bio-Rad, Hercules, CA, USA). Primers were synthesized by Life Technologies (Tokyo, Japan) (Table 1). The reaction conditions were 40 cycles of 95°C for 15 s and 60°C for 40 s. The dissociation curve for each sample was analyzed to verify the specificity of each reaction. Relative mRNA levels of target genes were determined with the ΔΔCt method and normalized to the expression of* ribosomal protein L32* (*RPL32*) (in vivo analysis) or* glyceraldehyde 3 phosphate dehydrogenase* (*GAPDH*) (in vitro analysis). There were no differences between results determined by the ΔΔCt and the standard curve methods (data not shown).

### 2.6. Protein Extraction and Western Blot Analysis

Tissue samples were homogenized in lysis buffer (50 mM HEPES, pH 7.4; 150 mM NaCl; 10 mM EDTA; 10 mM NaF; 10 mM Na_4_P_2_O_7_; 2 mM Na_3_VO_4_; 1% sodium deoxycholate; 1% Nonidet P-40; and 0.2% sodium dodecyl sulphate (SDS)) with protease inhibitor mix containing aprotinin, E-64, leupeptin hemisulfate monohydrate, bestatin, and pepstatin A (Nacalai Tesque, Kyoto, Japan) on ice. Homogenates were centrifuged at 1770 ×g and 4°C for 10 min and the supernatant was collected. Protein concentration was measured using a bicinchoninic acid protein assay kit (Thermo Fisher Scientific, Yokohama, Japan) and normalized to 2 *μ*g/*μ*L with SDS-polyacrylamide gel electrophoresis (PAGE) loading buffer (62.5 mM Tris-HCl, pH 6.8; 2% w/v SDS; 10% glycerol; 50 mM dithiothreitol; and 0.01% w/v Bromophenol Blue). Protein samples were resolved by SDS-PAGE (SuperSep Ace; Wako Pure Chemical Industries) and transferred to polyvinylidene difluoride membranes, which were blocked with Blocking One solution (Nacalai Tesque) for 1 h at room temperature. Membranes were probed overnight at 4°C with the following primary antibodies (all from Cell Signaling Technology, Danvers, MA, USA): rabbit anti-Akt (1 : 1,000), rabbit anti-phospho-Akt (Ser473; 1 : 1,000), rabbit anti-p70 S6 kinase (p70S6K) (1 : 1,000), rabbit anti-phospho-p70S6K (Thr389; 1 : 500), rabbit anti-glycogen synthase kinase (GSK) 3*β* (1 : 2,000), rabbit anti-phospho-GSK3*β* (Ser9; 1 : 2,000), rabbit anti-*β*-catenin (1 : 2,000), and anti-GAPDH (1 : 2,000). This was followed by incubation with horseradish peroxidase-conjugated donkey anti-rabbit IgG (1 : 20,000; GE Healthcare, Fairfield, CT, USA) for 1 h at room temperature. After repeated washes in TBS containing 0.05% Tween 20, membranes were incubated in Pierce Thermo Western Blotting Substrate (Thermo Fisher Scientific, Waltham, MA, USA) and protein bands were visualized by chemiluminescence on an LAS-3000 Mini system (Fujifilm, Tokyo, Japan). Images of each membrane were analyzed using National Institutes of Health ImageJ software (http://rsbweb.nih.gov/ij/) as previously described [21]. Mean intensity and standard deviation (SD) were calculated. *β*-catenin immunoreactivity was normalized to that of GAPDH.

### 2.7. Statistical Analysis

Data were analyzed with Student's *t*-test and are expressed as mean ± SD. *P* values < 0.05 were considered significant.

## 3. Results

### 3.1. FO Induces Skeletal Muscle Hypertrophy

**Figure 1:**
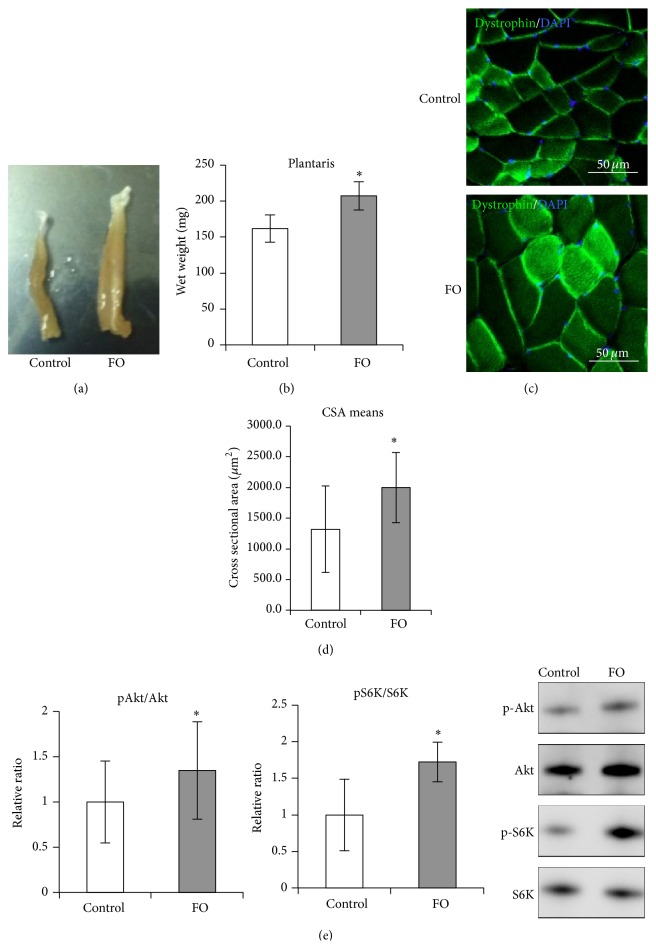
Skeletal muscle hypertrophy following functional overload. (a, b) Change in muscle weight following functional overload. A photograph of plantaris muscles isolated from control and FO groups (a) and a graph representing wet weight of plantaris in each group (b). (c, d) Change in cross-sectional area of plantaris following functional overload. Representative merged images of immunohistochemistry staining for Dystrophin (green) with DAPI from control and FO groups (c) and a graph plotting the means of cross-sectional area in each group (d) are shown. (e) Representations of Akt (Ser473) phosphorylation levels (left) and p70S6K (Thr389) phosphorylation levels (center), as detected by Western blot analysis. The typical blot patterns are described in the right panels. Phosphorylation levels were calculated to divide the signal of the phosphorylated form against the total protein expression for Akt or S6K. The relative ratio, normalized to the signal observed for the control group, is shown. All values are expressed as the mean ± SDM (*n* = 5). Significant differences: *∗*compared to control group (*P* < 0.05).

Rats were subjected to FO for 1 week to model resistance exercise. There were no differences in body mass between FO and control rats at the end of the experiment (data not shown). However, the wet weight of the plantaris muscles was significantly higher in FO than control rats (207.28 ± 19.7 mg versus 161.72 ± 19.3 mg) (Figures 1(a) and 1(b)). The cross-sectional area of the plantaris muscle also increased following FO (Figures 1(c) and 1(d)). In previous studies, FO resulted in the activation of Akt/mammalian target of rapamycin/p70S6K signaling, which increased cellular protein synthesis [22, 23]. Accordingly, Akt (Ser473) and S6K (Thr389) phosphorylation levels were higher in FO than in control rats (Figure 1(e)). These results indicate that FO reliably induces plantaris hypertrophy.

### 3.2. Functional Overload Facilitated Satellite Cell Activation and Proliferation

**Figure 2:**
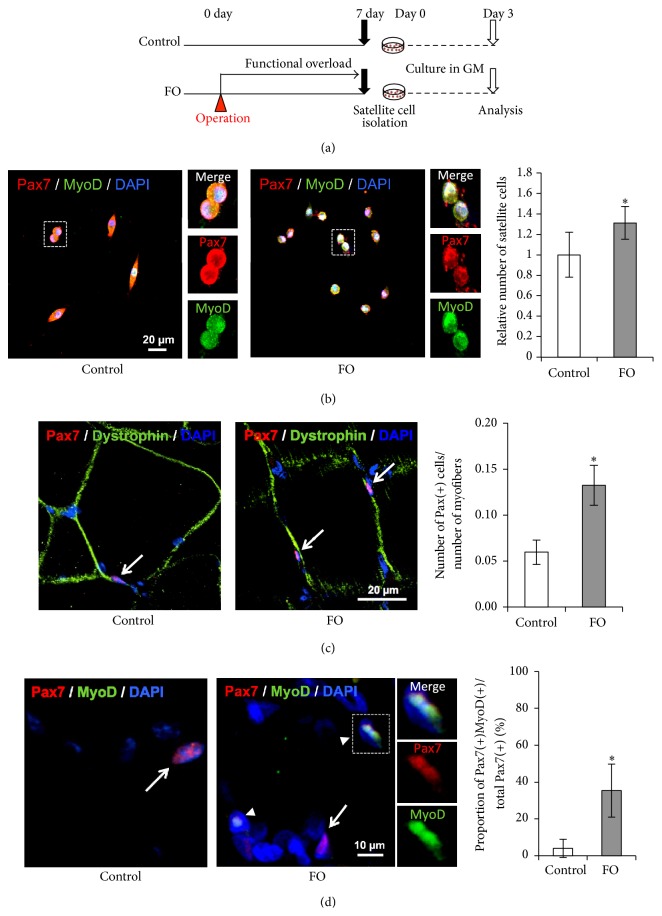
Satellite cell activation and proliferation following functional overload. (a) Schematic representation of the experimental design. FO group rats had a surgical operation to ablate synergistic muscles and received functional overload stimulation for 1 week. Satellite cells were isolated from plantaris of each group of rats 7 days after operation and were cultured in growth medium for 3 days. (b) Immunocytochemistry analysis of cultivated-satellite cells. Representative merged images of immunocytochemistry staining for Pax7 (red) and MyoD (green) with DAPI from control group (left panel) and FO group (right panel) are shown. Magnification of the area surrounded by the dotted square is shown in the right panels. Relative number, normalized to the number observed for the control group, of Pax7(+)MyoD(+) cells is shown in the right graph. (c, d) Immunohistochemistry analysis of satellite cells following functional overload. Representative merged images of immunohistochemistry staining for Pax7 (red) and Dystrophin (green) (c) or for Pax7 (red) and MyoD (green) (d) with DAPI from control group (left panel) and FO group (right panel) are shown. Magnification of the area surrounded by the dotted square is shown in the right panels. The proportion of Pax7(+) cells per myofibers (c) or the proportion of Pax7(+)MyoD(+) cells per total Pax7(+) cells (d) is shown in the right graphs. White arrows and arrowheads indicate Pax7(+) cells and Pax7(+)MyoD(+) cells, respectively. All values are expressed as mean ± SDM (*n* = 5). Significant differences: *∗* compared to control group (*P* < 0.05).

We examined the effects of FO on proliferation by immunocytochemical analysis of Pax7 and MyoD in a primary culture of satellite cells isolated from the plantaris muscle and cultured in growth medium for 3 days (Figure 2(a)). The number of Pax7^+^MyoD^+^ satellite cells increased >1.3-fold after FO (Figure 2(b)), indicating that proliferative capacity was enhanced.

Activated satellite cells express the myogenic regulatory factors (MRFs) Myf5 and MyoD, two key transcription factors for myogenic lineage progression and differentiation, in addition to the stem-cell-specific transcription factor Pax7 [7]. We investigated changes in the number as well as the character of satellite cells after FO by evaluating the expression of these markers. The number of Pax7^+^ cells was >2-fold higher in FO than in control rats (Figure 2(c)), consistent with a previous report [19]. Because Pax7^+^ cells include both quiescent and activated satellite cell populations, immunohistochemical staining for Pax7 and MyoD using plantaris cross sections was performed to determine how the satellite cell population changes after functional overload. Since positive Pax7 immunoreactivity is observed in both quiescent and activated satellite cells, we examined the coexpression of Pax7 and MyoD (a marker of activated satellite cells) in plantaris muscle cross sections to determine the identity of the satellite cell population after FO. Pax7^+^MyoD^+^ activated satellite cells were rarely observed in controls but were prevalent in FO rats; the number of Pax7^+^MyoD^+^ cells increased following FO by about 9-fold (Figure 2(d)). Taken together, these results suggest that resistance exercise induces satellite cell proliferation and activation.

### 3.3. Functional Overload Increased the Efficiency of Satellite Cell Differentiation

**Figure 3:**
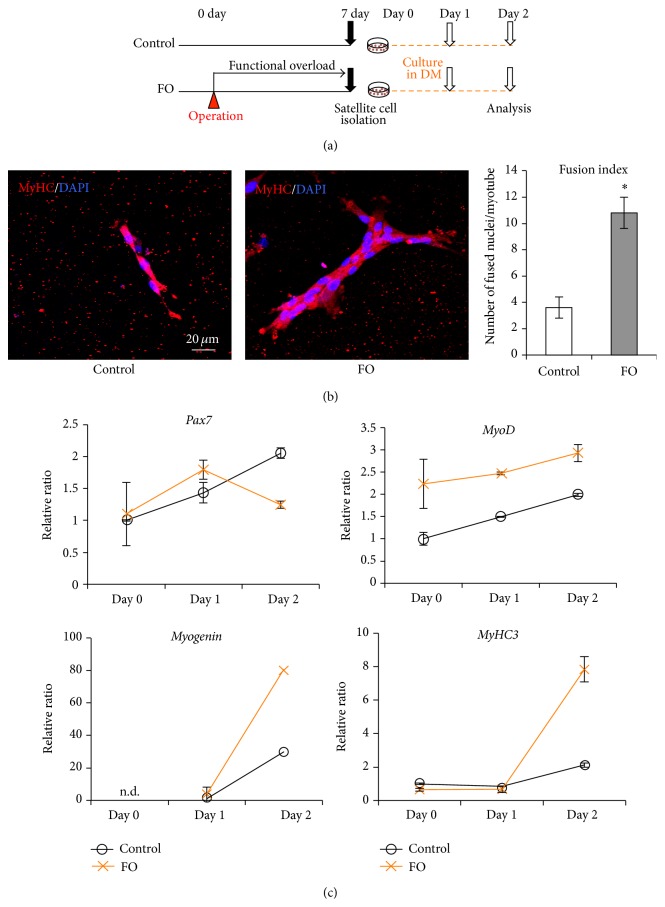
Acceleration of satellite cell differentiation following functional overload. (a) Schematic representation of the experimental design. FO group rats had a surgical operation to ablate synergistic muscles and received functional overload stimulation for 1 week. Satellite cells were isolated from plantaris of each group of rats 7 days after operation and were cultured in differentiation medium for 2 days. (b) Immunocytochemistry analysis of cultivated-satellite cells. Representative merged images of immunocytochemistry staining for MyHC (red) with DAPI from control group (left panel) and FO group (right panel) are shown. Fusion index, calculated to average the number of nuclei fused into a myotube, is shown in the right graph. (c) Expression profiles of stem cell markers in differentiation process of satellite cells. mRNA expression levels of *Pax7* (upper left), *MyoD* (upper right), *myogenin* (lower left), and *MyHC3* (lower right) in cultured cells were measured by qRT-PCR analysis on days 0, 1, and 2 (before and 1 and 2 days after inducing differentiation, resp.). In the graphs, black lines and orange lines represent control group and FO group, respectively. Target mRNA expression was normalized to that of* GAPDH* and then plotted as the expression ratio relative to the control group. All values are expressed as mean ± SDM (*n* = 5). Significant differences: *∗* compared to control group (*P* < 0.05). n.d.: not detectable.

Differentiating myoblasts fuse to generate myotubes. We investigated the effect of FO on the differentiation potential of satellite cells isolated from the plantaris muscle and cultured in differentiation medium for 2 days (Figure 3(a)). An immunocytochemical analysis of MyHC expression, which is indicative of newly generated myotubes, showed that myoblasts derived from FO satellite cells formed larger myotubes as compared to those from control cells; the fusion index (i.e., number of nuclei/myotube) was > 3-fold in the FO as compared to the control group (Figure 3(b)), indicating increased differentiation potential in cells from overloaded muscle.

The expression levels of *Pax7*,* MyoD*,* myogenin* (a differentiation marker), and* MyHC3* in isolated satellite cells were evaluated by qRT-PCR. RNA was collected from cells on days 0, 1, and 2 (before and 1 and 2 days after inducing differentiation, resp.) to detect changes in gene expression. Although* Pax7* expression in control cells increased over time, in FO cells the level increased on day 1 before returning to baseline on day 2 (Figure 3(c), upper left panel), suggesting that satellite cells derived from FO plantaris muscle committed to a myogenic lineage and thus downregulated* Pax7* expression at an earlier time point than control cells. Indeed,* MyoD* level increased over time in all cells but was consistently higher in FO than in control cells (Figure 3(c), upper right panel), suggesting that the majority of satellite cells derived from FO plantaris muscle were already activated when the cells were isolated.* Myogenin* and* MyHC3* were expressed at low levels on days 0 and 1; the levels were upregulated on day 2 in all cells but were significantly higher in FO as compared to control cells (Figure 3(c), lower panels), consistent with the observed increase in* MyoD* expression in the former. These results indicate that satellite cell differentiation and myotube formation were accelerated as a result of FO and that the decrease in* Pax7* expression and relatively high levels of* MyoD*,* myogenin*, and* MyHC3* may be responsible for efficient cell fusion in the generation of myotubes. Additionally, the increase in the mRNA expression of MRFs in FO cells suggests a regulatory link between the extracellular signal arising from FO and the transcriptional control of these myogenic genes (especially* MyoD*).

### 3.4. Overload-Induced Satellite Cell Activation Involves Notch, Wnt, and Sox

**Figure 4:**
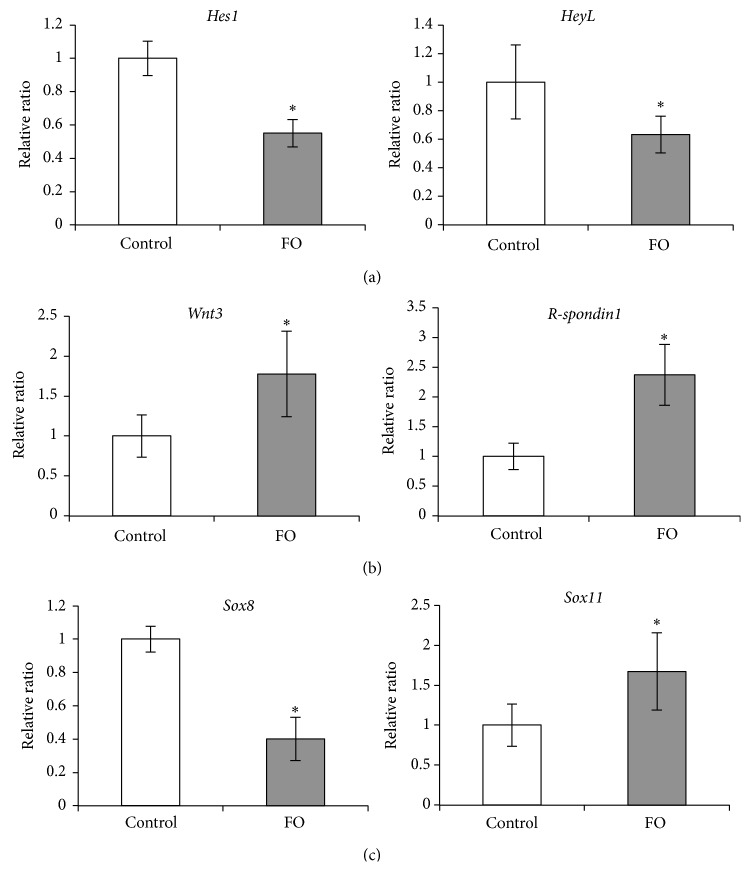
Changes in Notch, Wnt, and Sox expression following functional overload. (a, b, c) Expression levels of Notch signaling-related genes, Wnt signaling-related genes, and Sox genes. Amounts of *Hes1* and* HeyL* (a), *Wnt3* and *R-spondin1* (b), and* Sox8* and* Sox11* (c) mRNAs in the plantaris were measured by qRT-PCR analysis. Target mRNA expressions were normalized to that of* RPL32* and then plotted as the expression ratio relative to control group. All values are expressed as mean ± SDM (*n* = 5). Significant differences: *∗* compared to control group (*P* < 0.05).

The Notch and Wnt signaling pathways regulate satellite cell self-renewal and myogenesis during embryogenesis [24]. Binding of Notch receptors to their ligands releases the Notch intracellular domain, which is translocated into the nucleus and binds recombinant signal-binding protein for immunoglobulin *κ* J, thereby activating the transcription of target genes such as those belonging to the Hes and Hey families [25–27]. These basic helix-loop-helix (bHLH) repressors form inactive Hes/MyoD or Hey/MyoD heterodimers to inhibit MyoD expression in quiescent satellite cells, [28, 29], thereby preventing their differentiation into myoblasts. We assessed the expression of *Hes1* and* HeyL* in plantaris muscle by qRT-PCR and found that the transcript levels were significantly lower in FO than in control rats (Figure 4(a)), suggesting that resistance exercise induces a shift in satellite cells from a quiescent to an activated state by blocking Notch signaling.

Wnts are secreted proteins that bind to Frizzled receptors in the plasma membrane [30]. This stabilizes *β*-catenin, which forms a complex with T cell factor (TCF)/leukocyte enhancer factor (LEF) that is translocated into the nucleus and activates the transcription of target genes [31, 32]. Wnt signaling regulates myogenesis via modulation of MRF expression [24], and it was recently found that the Wnt activator R-spondin induces myogenic differentiation of satellite cells [33]. We examined whether FO affects Wnt signaling in skeletal muscle by qRT-PCR analysis of plantaris muscle samples. *Wnt3* and* R-spondin1* mRNA levels were increased in FO as compared to control rats (Figure 4(b)), suggesting the activation of Wnt signaling by resistance exercise that caused a shift in satellite cells from a quiescent to an activated state.

The Sox family comprises high mobility group-box transcription factors that are involved in development and differentiation of various tissues. Sox8 and Sox9 are simultaneously downregulated in satellite cells during differentiation, and their overexpression inhibits myotube formation and leads to a reduction in the expression of MyoD and myogenin, suggesting that these factors negatively regulate satellite cell differentiation to maintain a pool of self-renewing progenitors [34]. Additionally, Sox11 is expressed in differentiated myotubes and may positively regulate satellite cell differentiation [34]. We found that* Sox8* transcript was decreased while that of* Sox11* was increased in FO as compared to control rats (Figure 4(c)), as determined by qRT-PCR. Taken together, our data suggest that resistance exercise induces satellite cell activation and myogenesis via regulation of Notch and Wnt signaling pathways and modulation of* Sox8* and* Sox11* levels.

## 4. Discussion

The present study investigated changes in satellite cell character induced by resistance exercise and the molecular mechanisms underlying their activation. We found that FO, an experimental model of resistance exercise, enhanced satellite cell proliferation and differentiation, effects that were likely exerted via regulation of Notch and Wnt signaling pathways.

Physical exercise has various physiological effects, including a reduction in body mass, increased maximum oxygen uptake, and metabolic improvements and an increased number of satellite cells in skeletal muscle [35–37], although the mechanistic basis for the enhancement of satellite cell proliferation and differentiation has not been previously reported. We confirmed in our model that satellite cells derived from overloaded muscles had higher proliferative capacity and differentiation potential as compared to those derived from control muscles. On the other hand, one study reported that satellite cells derived from nonloaded mice by hindlimb suspension decreased differentiation potential [38]. These findings suggest that externally applied mechanical stimuli, including physical exercise, can modulate skeletal muscle satellite cell properties. The bHLH transcription factor MyoD induces myogenic differentiation by forming heterodimers at E-box regulatory sequences of muscle-specific genes such as* myogenin*,* actin α1*,* myocyte enhancer factor 2s*,* troponin c2*,* i2*, and* t3* (*Tnnc2*,* Tnni2*, and* Tnnt3)*, among others [39, 40]. We found that satellite cells derived from overloaded muscles expressed high levels of* MyoD*, which was associated with increased proliferation and differentiation. It was recently demonstrated that exercise induces the chromatin remodeling at the* MyoD* promoter and stimulates transcription [13]. Therefore, resistance exercise likely promotes satellite cell proliferation and differentiation by inducing the upregulation of* MyoD*.

Notch and Wnt signaling pathways have been shown to regulate satellite cell fate determination and proliferation. Notch signaling, which is activated by physical exercise [8], suppresses myogenic differentiation via inhibition of* MyoD* expression in satellite cells. However, we found that the expression of the Notch targets* Hes1* and* HeyL* decreased following 1 week of FO. One possible explanation for this is that exercise-induced Notch activation is a transient response that returns to the baseline 18 h later [41]. Another study showed that the levels of* HeyL* and* Delta4*, which is a principal ligand of the Notch signaling pathway, were downregulated in adult mice following 4 weeks of wheel running [13]. Moreover, inhibiting Notch signaling using a pharmacological *γ*-secretase inhibitor induced myotube hypertrophy [42]. Thus, exercise-induced changes in satellite cell properties are likely modulated by Notch signaling, although the detailed mechanism has yet to be elucidated.

Wnt signaling plays essential roles in embryonic development but also in the maintenance of adult stem cells in various tissues. In adult neurogenesis, astrocyte cells surrounding adult neural stem cells secrete Wnt3, which triggers neurogenesis by inducing the expression of the bHLH transcription factor NeuroD [43–45]. Since Wnt3 expression in the adult brain can be altered by external stimuli such as exercise [46], stem cell niches act as sensors that regulate adult neural stem cell behavior. In adult skeletal muscle,* Wnt3* and* R-spondin1* expression was increased along with satellite cell activation by resistance exercise. We surmised that Wnt-induced* MyoD* expression in satellite cells caused their shift from a quiescent to an activated, proliferative state in rats subjected to FO, consistent with the findings of a previous study [13].

Sox genes are essential for the development and differentiation of various tissues as well as fate determination in satellite cells [34]. We found that the switch in satellite cells from quiescent state to activated state occurred in conjunction with* Sox8* downregulation, suggesting that Sox8 negatively regulates satellite cell activation. During central nervous system development, Sox2 blocks neurogenesis in embryonic stem cells [47]. However, Wnt activation along with the removal of Sox2 triggers* NeuroD* expression and neurogenesis. This regulatory mechanism depends on overlapping Sox2 and TCF/LEF binding sites in the* NeuroD1* promoter [44], implying that neuronal differentiation occurs via crosstalk between Sox2 and Wnt signaling. We propose that the expression of MyoD—another bHLH transcription factor—in satellite cells is regulated by a similar mechanism and that Sox8 regulates myogenic differentiation of these cells via modulation of the Wnt signaling pathway, although a more detailed characterization of this interaction is required.

## 5. Conclusions

The findings of study revealed the effects of resistance exercise on the regulation of satellite cells properties, including an increase in cell number and enhanced differentiation potential resulting in skeletal muscle hypertrophy. These effects were associated with the downregulation of Notch and upregulation of Wnt signaling and likely involve modulation by transcription factors of the Sox family. Our results suggest that resistance exercise can be effective in preventing the progression of sarcopenia. However, further studies investigating the factors that link Notch and Wnt signaling pathways to satellite cell activation during exercise are needed for a more complete understanding of the functions and intrinsic ability of satellite cells in adult myogenesis.

## Figures and Tables

**Table 1:** Primer sequences for qRT-PCR.

Target gene	Sequence (5′-3′)		Product length
*GAPDH*	Forward	GTATGTCGTGGAGTCTACTG	157 bp
	Reverse	CTTGAGGGAGTTGTCATATTTC	

*RPL32*	Forward	AGATTCAAGGGCCAGATCCT	196 bp
	Reverse	CTACGAAGGCTTTTCGGTTC	

*Pax7*	Forward	AGTGAGTTCGATTAGCCGAG	153 bp
	Reverse	GAGCCTTCATCAAGACGGTT	

*MyoD*	Forward	GCAAGCGCAAGACCACTAAC	172 bp
	Reverse	TCAATGTAGCGGATGGCGTT	

*Myogenin*	Forward	TCAACCAGGAGGAGCGCGAT	208 bp
	Reverse	ATGCTGTCCACGATGGACGT	

*MyHC3*	Forward	TGCTGTGCTGTACAACCTCA	201 bp
	Reverse	AGCATGAACTGGTAGGCGTT	

*Hes1*	Forward	CAACACGACACCGGACAAAC	159 bp
	Reverse	TTGGAATGCCGGGAGCTATC	

*HeyL*	Forward	AGCCAGCTTTCGCCATGAAG	179 bp
	Reverse	GCGCCGTTTCTCTATGATCC	

*Wnt3*	Forward	TTGCGTCTTCCACTGGTGCTGCTA	206 bp
	Reverse	AGCTGGCAATCGTCCTTGCTCCTT	

*R-spondin1*	Forward	CGCCTGGATACTTTGATGCC	115 bp
	Reverse	AAGCCCTCCTGACACTTGGT	

*Sox8*	Forward	TATGGAGGCGCTTCCTACTC	141 bp
	Reverse	CAGCTGCTCCGTCTTGATAT	

*Sox11*	Forward	GCGGTCAGGATAAAGAGGATG	200 bp
	Reverse	AAAGGAAGGGAAGAGTGGGGA	
